# HMGA1a Induces Alternative Splicing of the *Estrogen Receptor-*α*lpha* Gene by Trapping U1 snRNP to an Upstream Pseudo-5′ Splice Site

**DOI:** 10.3389/fmolb.2018.00052

**Published:** 2018-06-08

**Authors:** Kenji Ohe, Shinsuke Miyajima, Tomoko Tanaka, Yuriko Hamaguchi, Yoshihiro Harada, Yuta Horita, Yuki Beppu, Fumiaki Ito, Takafumi Yamasaki, Hiroki Terai, Masayoshi Mori, Yusuke Murata, Makito Tanabe, Ichiro Abe, Kenji Ashida, Kunihisa Kobayashi, Munechika Enjoji, Takashi Nomiyama, Toshihiko Yanase, Nobuhiro Harada, Toshiaki Utsumi, Akila Mayeda

**Affiliations:** ^1^Department of Pharmacotherapeutics, Faculty of Pharmaceutical Sciences, Fukuoka University, Fukuoka, Japan; ^2^Department of Breast Surgery, Fujita Health University, Toyoake, Japan; ^3^Department of Endocrinology and Diabetes Mellitus, Faculty of Medicine, Fukuoka University, Fukuoka, Japan; ^4^Department of Endocrinology and Diabetes Mellitus, Fukuoka University Chikushi Hospital, Chikushino, Japan; ^5^Department of Medicine and Bioregulatory Science, Graduate School of Medical Sciences, Kyushu University, Fukuoka, Japan; ^6^Department of Biochemistry, Fujita Health University, Toyoake, Japan; ^7^Division of Gene Expression Mechanism, Institute for Comprehensive Medical Science, Fujita Health University, Toyoake, Japan

**Keywords:** estrogen receptor alpha, HMGA1a, alternative splicing, U1 snRNP, breast cancer

## Abstract

**Objectives:** The high-mobility group A protein 1a (HMGA1a) protein is known as a transcription factor that binds to DNA, but recent studies have shown it exerts novel functions through RNA-binding. We were prompted to decipher the mechanism of HMGA1a-induced alternative splicing of the estrogen receptor alpha (ERα) that we recently reported would alter tamoxifen sensitivity in MCF-7 TAMR1 cells.

**Methods:** Endogenous expression of full length ERα66 and its isoform ERα46 were evaluated in MCF-7 breast cancer cells by transient expression of HMGA1a and an RNA decoy (2′-O-methylated RNA of the HMGA1a RNA-binding site) that binds to HMGA1a. RNA-binding of HMGA1a was checked by RNA-EMSA*. In vitro* splicing assay was performed to check the direct involvement of HMGA1a in splicing regulation. RNA-EMSA assay in the presence of purified U1 snRNP was performed with psoralen UV crosslinking to check complex formation of HMGA1a-U1 snRNP at the upstream pseudo-5′ splice site of exon 1.

**Results:** HMGA1a induced exon skipping of a shortened exon 1 of ERα in *in vitro* splicing assays that was blocked by the HMGA1a RNA decoy and sequence-specific RNA-binding was confirmed by RNA-EMSA. RNA-EMSA combined with psoralen UV crosslinking showed that HMGA1a trapped purified U1 snRNP at the upstream pseudo-5′ splice site.

**Conclusions:** Regulation of ERα alternative splicing by an HMGA1a-trapped U1 snRNP complex at the upstream 5′ splice site of exon 1 offers novel insight on 5′ splice site regulation by U1 snRNP as well as a promising target in breast cancer therapy where alternative splicing of ERα is involved.

## Introduction

Cancer-associated alternative splicing has been extensively studied in various steps from tumor initiation to progression and metastasis (Oltean and Bates, 2014; Chen and Weiss, 2015). These cancer-associated alternative splicing events are aberrantly regulated by multifunctional bona fide splicing factors [serine/arginine-rich (SR) proteins and heterogeneous nuclear ribonucleoproteins (hnRNPs)] and tissue-specific RNA-binding proteins (David and Manley, 2010) possessing oncogenic potential *per se* (Karni et al., 2007). In addition, several recent reports have evoked attention on the importance of alternative splicing of the clinically important biomarkers in breast cancer (Inoue and Fry, 2015), and the deregulation and involvement of splicing factors in breast cancer-associated alternative splicing (Silipo et al., 2015).

Estrogen receptor alpha (ERα) is an important biomarker as well as the key factor in estrogen-dependent growth of breast cancer. We have recently found that high-mobility group A protein 1a (HMGA1a) is involved in alternative splicing of ERα (Ohe et al., 2018). The resulting transcript, called ERα46 (Flouriot et al., 2000), is known for its function in partially inhibiting mitogenic activity of full length ERα (Penot et al., 2005).

HMGA1a is originally known as HMGI (Lund et al., 1983), a nonhistone DNA-binding protein of the UBF/HMG family, and an oncoprotein which induces cancerous transformation (Reeves, 2010). In addition to its DNA-binding properties exerted by its three AT hooks, we have shown HMGA1a protein binds to a specific RNA sequence of 5′-GC(U)GCUACAAG-3′, adjacently upstream the authentic 5′ splice site of exon 5 in the *Presenilin-2* (*PS2*) gene, interacts with U1-70K (Manabe et al., 2003), and traps U1 snRNP to this 5′ splice site to inhibit normal dissociation of U1 snRNP from the spliceosome and induces aberrant splicing of *PS2* exon 5 (Ohe and Mayeda, 2010). This effect was also found in HIV-1 splice site regulation (Tsuruno et al., 2011). A report of an aptamer search for the second AT hook motif of HMGA1 proteins shows binding to a G-rich motif of 5′-GGGGNGNGGNUGGGGNGG-3′ (Maasch et al., 2010). Another study shows HMGA1a binds the second loop of 7SK RNA (5′-UGCGC-3′) (Eilebrecht et al., 2011b). Recently, it has been reported that AT hook proteins such as HMGA1a bind RNA with one order higher affinity than DNA (Filarsky et al., 2015). Proteomics studies have reported that HMGA1a interacts with mRNA processing proteins by GST pull-down and farwestern experiments (Sgarra et al., 2005, 2008; Pierantoni et al., 2007). Thus, the recent description of the role of HMGA1a in RNA metabolism in addition to its role as a DNA-binding transcription factor make it more multi-functional protein than previously believed.

## Materials and methods

### Plasmids, antisense oligonucleotides, 2′-O-methyl RNAs, recombinant protein, purified U1 snRNP

Plasmids were constructed as indicated below.

Plasmid for CDC-ERα pre-mRNA was constructed by nearby exon and intron sequences of the splice sites of ERα exon 1 attached to the 5′ ends of the primers which were used to amplify CDC14-15 (chicken delta crystallin exon 14-15) (Sawa et al., 1988; Kataoka et al., 2000) by PCR. The primers used for this PCR reaction were δESRexon1_FW and δESRexon1_RV. The PCR fragment was digested with Hind III and self-ligated resulting in pSP64-CDC-ERα with shortened ERα exon 1 (58 bp) and flanking intron sequence placed in the intron of CDC14-15. pSP64-CDC-ERα plasmid was linearized using Sma1 for RNA synthesis.

The riboprobe template plasmids for ERαEx1-5SS_wt, ERαEx1-5SS_HMGBSmut, ERαEx1-5SS_A5SSmut, ERαEx1-5SS_P5SSmut were constructed by PCR using pSP64 as template and digested by HindIII. The forward primers were ERαEx1-5SS_wt_FW, ERαEx1-5SS_HMGBSmut_FW, αEx1-5SS_A5SSmut_FW, ERαEx1-5SS_P5SSmut_FW, and the reverse primer is ERαEx1-5SS_RV (Table 1). The HindIII-digested PCR products were self-ligated at the Hind III site to obtain the pSP64-ERαEx1-5SS_wt, pSP64-ERαEx1-5SS_HMGBSmut, pSP64-ERαEx1-5SS_A5SSmut, pSP64-ERαEx1-5SS_P5SSmut plasmids. These plasmids were linearized using EcoRI.

**Table 1:** Oligonucleotides used in this study.

**δESRexon1_FW**	**5^′^- CGC*AAGCTT*GCGGCTACACGGTGCGCGAGGCCGGCCCGCCGGCATTCTACAGGTACCCGCgtccccagggagcagcaaa-3^′^**
**δESRexon1_RV**	**5^′^- CGC*AAGCTT*GCGGGCCACCTGGAAAAAGAGCACAGCCCGAGGTTAGAGGCtgtcctccaaccacattaaataatgagaa-3^′^**
** FW:forward, RV:reverse**	** Shortened ERα exon 1 (bold) and flanking intron sequence in upper case (Hind III is italicized) and CDC14-15 intron sequence is in**
	** lower case**
ERαEx1-5SS_wt_FW	5′- ATAC*AAGCTT*CCCAGCGGCTACACG**GT**GCGCGAGGCCGGCCCGCCGGCATTCTACAGgtacccgcgGAATTCGTAATCATGGTCATAGCTG−3′
ERαEx1-5SS_HMGBSmut_FW	5′- ATAC*AAGCTT*CCCAGCCGAGATACG**GT**GCGCGAGGCCGGCCCGCCGGCATTCTACAGgtacccgcgGAATTCGTAATCATGGTCATAGCTG−3′
ERαEx1-5SS_A5SSmut_FW	5′- ATAC*AAGCTT*CCCAGCGGCTACACG**GT**GCGCGAGGCCGGCCCGCCGGCATTCTACTCctacccgcgGAATTCGTAATCATGGTCATAGCTG−3′
ERαEx1-5SS_P5SSmut_FW	5′- ATAC*AAGCTT*CCCAGCGGCTACACTCCGCGCGAGGCCGGCCCGCCGGCATTCTACAGgtacccgcgGAATTCGTAATCATGGTCATAGCTG−3′
ERαEx1-5SS_RV	5′-CCC*AAGCTT*GTATTCTATAGTGTCACCTAAATCGTATGT-3′
FW:forward, RV:reverse	Hind III site is italicized, HMGA1a binding sequence is underlined, intron sequence in lower case, GU of pseudo 5′ splice site is in
	bold, mutated nucleotides are in red.
αP5′SS	5′-CTCGCGCACCGT-3′
αA5′SS	5′-CGCGGGTACCTG-3′
αU1	5′-CTGCCAGGTAAGTAT-3′
αU2	5′-AGGCCGAGAAGCGAT-3′
cont	5′-AGGGAGTATGTGAATGCC-3′

All of the linearized plasmids were transcribed with SP6 RNA polymerase in the presence of [α-^32^P]UTP. The sequences for antisense oligonucleotides used for RNase H cleavage of the upstream pseudo-5′ splice site (αP5′SS) and authentic-5′ splice site (αA5′SS) and the ones used for digesting 5′ ends of U1 (αU1) and U2 (αU2) snRNA, as well as the control oligonucleotide (cont), are shown in Table 1. The sequences for 2′-O-methyl RNA_HMGA1a_mut and 2′-O-methyl RNA_HMGA1a_wt that were purchased from Fasmac Co., Ltd. are also shown in Table 2. Recombinant HMGA1a was a kind gift from K. Kanameki and Y. Muto (Musashino, Tokyo, Japan) purified from *E. coli* and microdialyzed in buffer D (Mayeda and Krainer, 1999b) for *in vitro* splicing analyses. Purified U1 snRNP was kindly provided by the laboratory of R. Lührmann (Göttingen, Germany).

**Table 2:** 2′-O-methyl RNA used in this study.

**2^′^-O-methyl RNA_HMGA1a_mut**	**5^′^-CGCCGAGAUCAG-3^′^**
2′-O-methyl RNA_HMGA1a_wt	5′-CGCUGCUACAAG-3′

### Electrophoretic mobility shift assays (EMSA)

EMSA was performed as previously described (Ohe and Mayeda, 2010) with the following minor modifications. Each reaction mixture was incubated at 30°C for 15 min and RNA-protein complexes were analyzed by 5% polyacrylamide gel electrophoresis (PAGE) (acrylamide: bisacrylamide ratio 30:1 [wt/wt]) at 4°C.

### *In vitro* splicing, psoralen UV crosslinking assay

*In vitro* splicing was performed as described (Ohe and Mayeda, 2010), with minor modifications. ^32^P-labeled pre-mRNA (~20 fmol) was incubated at 30°C for 2 h in a 12.5 μl reaction mixture containing 3 mM ATP, 20 mM creatine phosphate, 20 mM HEPES-NaOH (pH 7.3), 3.5 mM MgCl_2_, 2% (wt/vol) low-molecular-weight polyvinyl alcohol (Sigma), and 3.5 μl of HeLa cell nuclear extract (CilBiotech). Recombinant protein was added first to the probe prior adding nuclear extract. Where indicated 20 pmol of SRSF1 was added. Psoralen-mediated UV crosslinking assay was performed as previously described fixing the incubation time at 5 min (Ohe and Mayeda, 2010).

### Immunoblot assays

MCF-7 cells were washed twice with PBS and resuspended in 1 ml TRIzol reagent (Invitrogen). After passing through a 25 G needle five times, protein was purified from the interphase and organic phase by precipitating with 6 V of a solution for precipitating protein (50% Ethanol, 24.5% Acetone, 24.5% Methanol, 1% distilled water). Twenty micrograms of protein was boiled in sample buffer and separated by 10% sodium dodecyl sulfate-PAGE (SDS-PAGE), transferred to nitrocellulose membrane and analyzed by an ERα antibody (HC-20) (Santa-Cruz, sc-543) which recognizes the C-terminus of the protein or HMGA1a antibody (FL95; Santa Cruz). Anti-rabbit immunoglobulin G conjugated to alkaline phosphatase (Promega) was used as secondary antibody and detected by BCIP (5-bromo-4-chloro-3-indolylphosphate) (Promega).

### Statistical analysis

Data are presented as mean ± SD. Group differences were analyzed by Students *t-*test using Microsoft Excel.

## Results

### HMGA1a binds a sequence in *ERα* exon 1

In our previous reports, we showed that HMGA1a binds to RNA in sequence-specific manner (Manabe et al., 2003, 2007; Ohe and Mayeda, 2010). Here, we searched for other binding sites in other diseases. Since HMGA1a has been intensely studied in breast cancer as a transcription factor and that its expression correlates with its malignant potential, we were motivated to seek whether its RNA-binding characteristics have any involvement in the development of the disease. Accordingly, we found a candidate HMGA1a RNA-binding site in the *estrogen receptor alpha* (*ER*α) gene. It functioned in MCF-7 cells, induced estrogen-dependent growth in these cells as well as nude mice, and tamoxifen-resistant MCF-7 TAMR1 cells were sensitized to tamoxifen (Ohe et al., 2018). Here we show the *in vitro* analyses of HMGA1a-trapped U1 snRNP at the upstream pseudo 5′ splice site adjacent the HMGA1a RNA-binding site in *ER*α exon1, which is located downstream of several non-coding exons.

**Figure 1:**
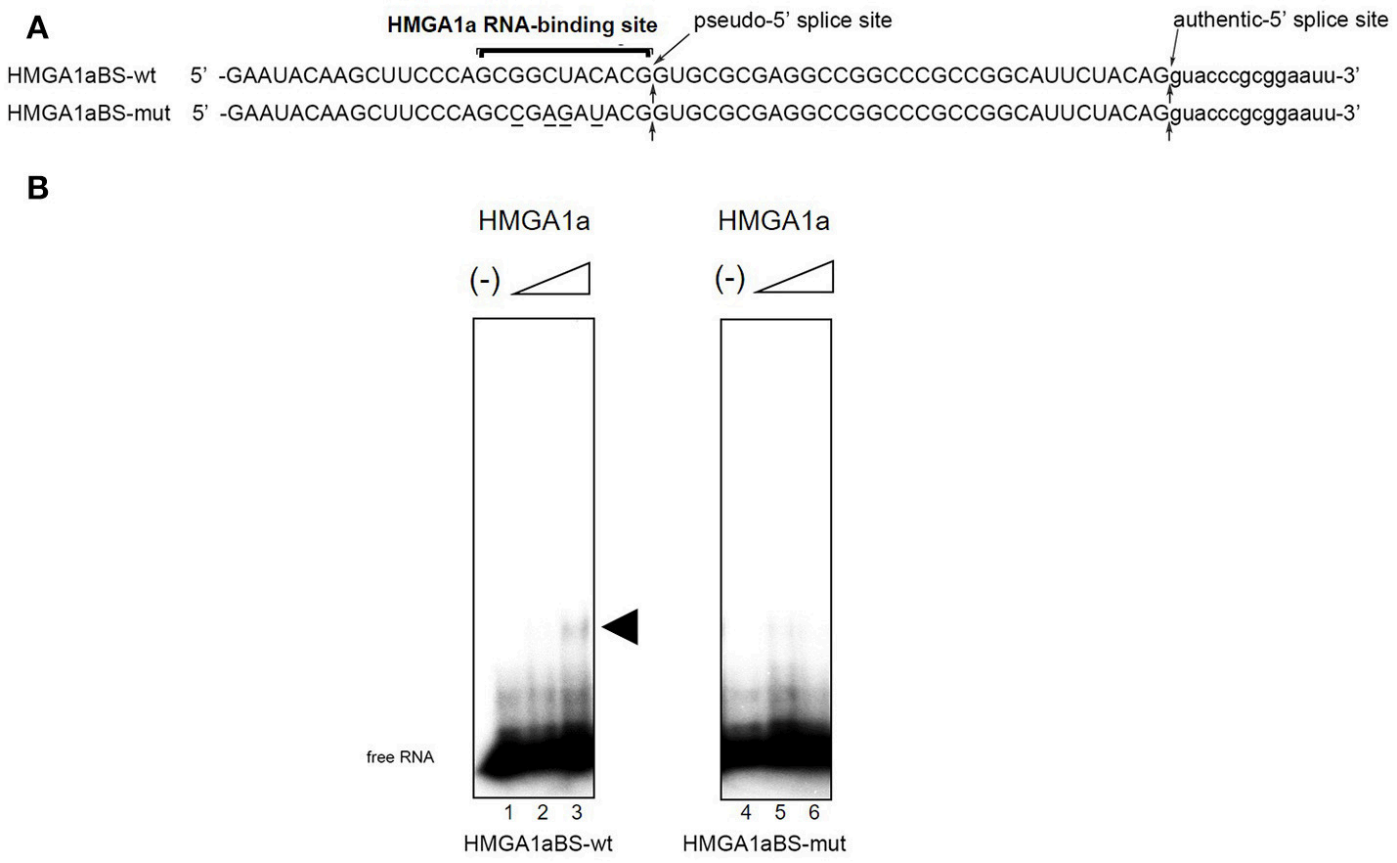
HMGA1a regulates alternative splicing of the *ER*α gene in MCF-7 cells. **(A)** Sequences of the HMGA1a RNA-binding sequence in *ER*α exon 1a is indicated (HMGA1a_BSwt). Arrows show the pseudo-5′ splice site and authentic 5′ splice site. Mutated bases are underlined (HMGA1a_BSmut). **(B)** HMGA1a binding to ^32^P-labeled RNAs were analyzed by RNA-EMSA. Increasing amounts of recombinant HMGA1a (0, 191, 383 ng in 15 μl) are indicated by minus signs and triangles. The shifted HMGA1a bound ^32^P-labeled RNA is indicated by arrowhead.

The candidate HMGA1a RNA-binding site was located 33 nucleotides upstream the authentic 5′ splice site (Figure 1A: HMGA1aBS-wt) of *ER*α exon 1. The 5′ splice site score of the adjacent pseudo-5′ splice site (MaxEnt: 8.67; Yeo and Burge, 2004) is comparable to the authentic 5′ splice site (MaxEnt: 8.63) of this exon. The HMGA1a RNA-binding candidate sequence is 5′-GC*G*GCUACA*C*G-3′, a two-base mismatch of the original one we found previously, 5′-GCUGCUACAAG-3′ (Manabe et al., 2003, 2007; Ohe and Mayeda, 2010) (mismatch underlined). RNA electrophoretic mobility shift assay (EMSA) (Figure 1B).

### HMGA1a induces exon skipping of a shortened ERα exon 1 *in vitro*

**Figure 2:**
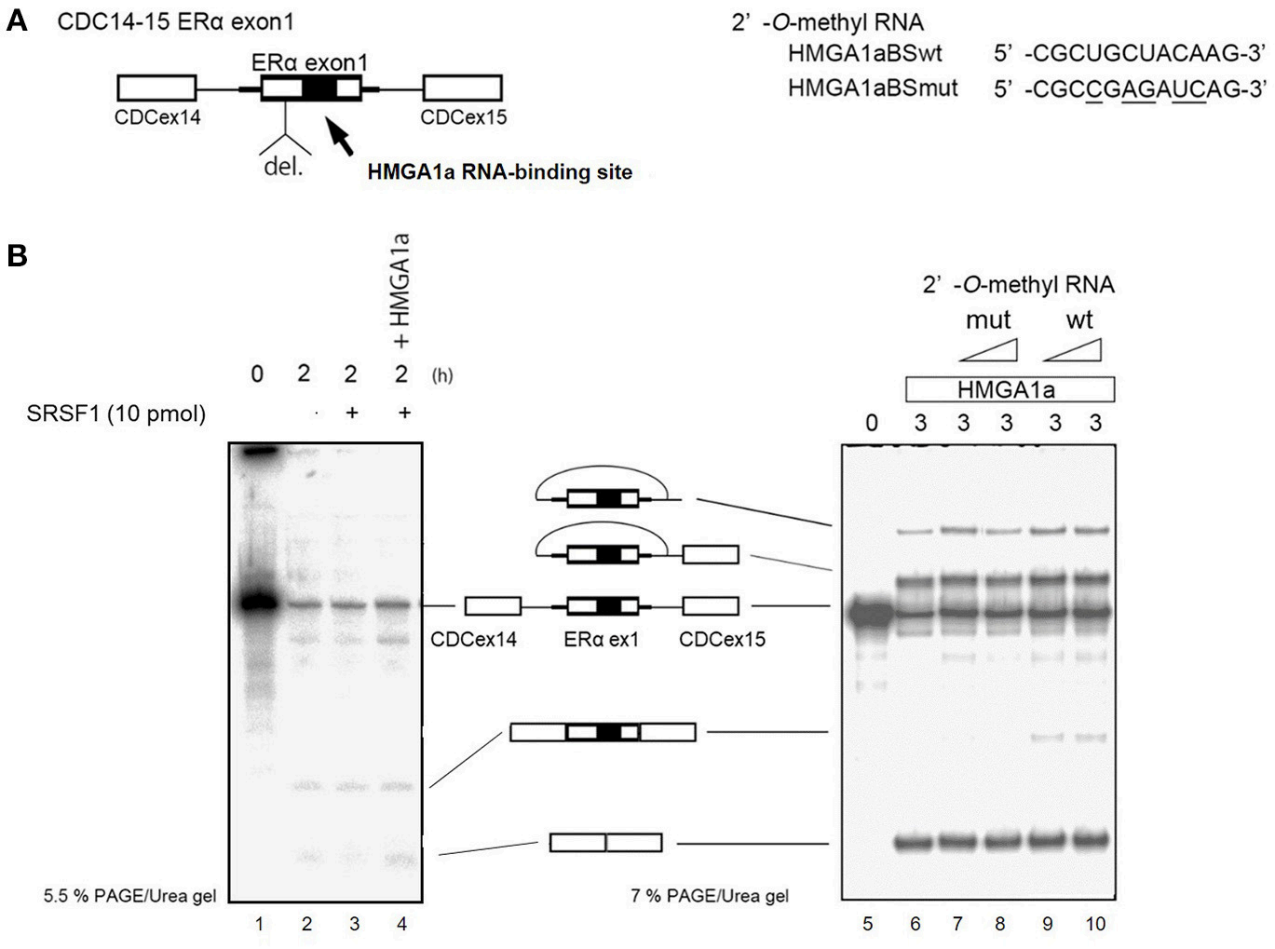
HMGA1a regulates alternative splicing of ERα exon 1 *in vitro*. **(A)** Scheme shows CDC-ERα pre-mRNA: shortened [indicated as del. (deletion of nucleotides)] ERα exon 1 and flanking intron sequence (bold line) inserted in the intron (thin line) of CDC14-15 pre-mRNA. The 2′-*O*-methyl RNA used in **(B)** are shown on right side. **(B)** (left panel) HMGA1a induces exon skipping of CDC-ERα (lane 1). SRSF1 was added as indicated (lanes 3,4). Two hundred and eighty-seven nanograms in 12.5 μl reaction of recombinant HMGA1a was added in lane 4. **(B)** (right panel) 2′-*O*-methyl RNA of HMGA1a RNA-binding sequence increases exon inclusion. Fifty and one hundred picomoles of 2′-*O*-methyl RNA are indicated as triangles. White arrowhead shows increase of exon inclusion by HMGA1a RNA-binding sequence 2′-*O*-methyl RNA.

ERα46 has been reported to be expressed through differential regulation of translation (Barraille et al., 1999), thus we needed to exclude this possibility by testing HMGA1a in an *in vitro* splicing assay where translational regulation is not observed. A heterologous pre-RNA transcript with the splice sites and flanking sequences of *ER*α exon 1 were inserted in the intron of a conventional splicing substrate, CDC14-15 (Sawa et al., 1988; Kataoka et al., 2000), designated CDC-ERα. Exon 1 was shortened for the limitations of length in splicing RNA *in vitro* (Mayeda and Krainer, 1999a). The RNA transcribed from CDC-ERα includes the HMGA1a RNA-binding sequence in a shortened ERα exon1 with flanking intron sequences inserted in intron between CDC exon 14 and 15 (Figure 2A). The splicing recapitulated that of cultured MCF-7 cells (Ohe et al., 2018), except that CDC-ERα showed exon skipping using HeLa nuclear extract in our *in vitro* splicing assay (Figure 2B, lane 2), thus we added SRSF1 and exon inclusion was observed (Figure 2B, lane 3). When HMGA1a was added to this reaction, an increase of exon exclusion was observed (Figure 2B, lane 4). Since HMGA1a is known to be highly expressed in HeLa cells, it is possible that the endogenous HMGA1a in HeLa nuclear extract induced exon skipping in our *in vitro* splicing assays before SRSF1 was added (Figure 2B, lane 2). Next, in order to observe the decoy effect of PS2 HMGA1a RNA-binding sequence, we extended the splicing reaction to 3 h without adding SRSF1. In this condition, no exon inclusion of CDC-ERα was observed (Figure 2B, lane 6). To confirm whether this exon skipping event was due to RNA-binding of HMGA1a, 2′-*O*-methyl RNA of the PS2 HMGA1a RNA-binding sequence (Manabe et al., 2007) was added to the reaction. While 2′-*O*-methyl RNA of mutant HMGA1a RNA-binding sequence showed no exon inclusion at 8 and 16 μM (Figure 2B, lane 7,8), 2′-*O*-methyl RNA of wild-type sequence showed clear exon inclusion at the same concentration (Figure 2B, lane 9,10). We believe that this induction of exon inclusion is significant in such limited exon inclusion conditions of this pre-mRNA substrate *in vitro*. The decoy effect of PS2 HMGA1a RNA-binding sequence was observed to inhibit alternative splicing induced by endogenous HMGA1a protein of MCF-7 cells (Ohe et al., 2018). Here we focus on HMGA1a-induced exon skipping of CDC-ERα and conducted further experiments to decipher the mechanism.

### HMGA1a anchors U1 snRNP to the upstream pseudo-5′ splice site of ERα exon 1

**Figure 3:**
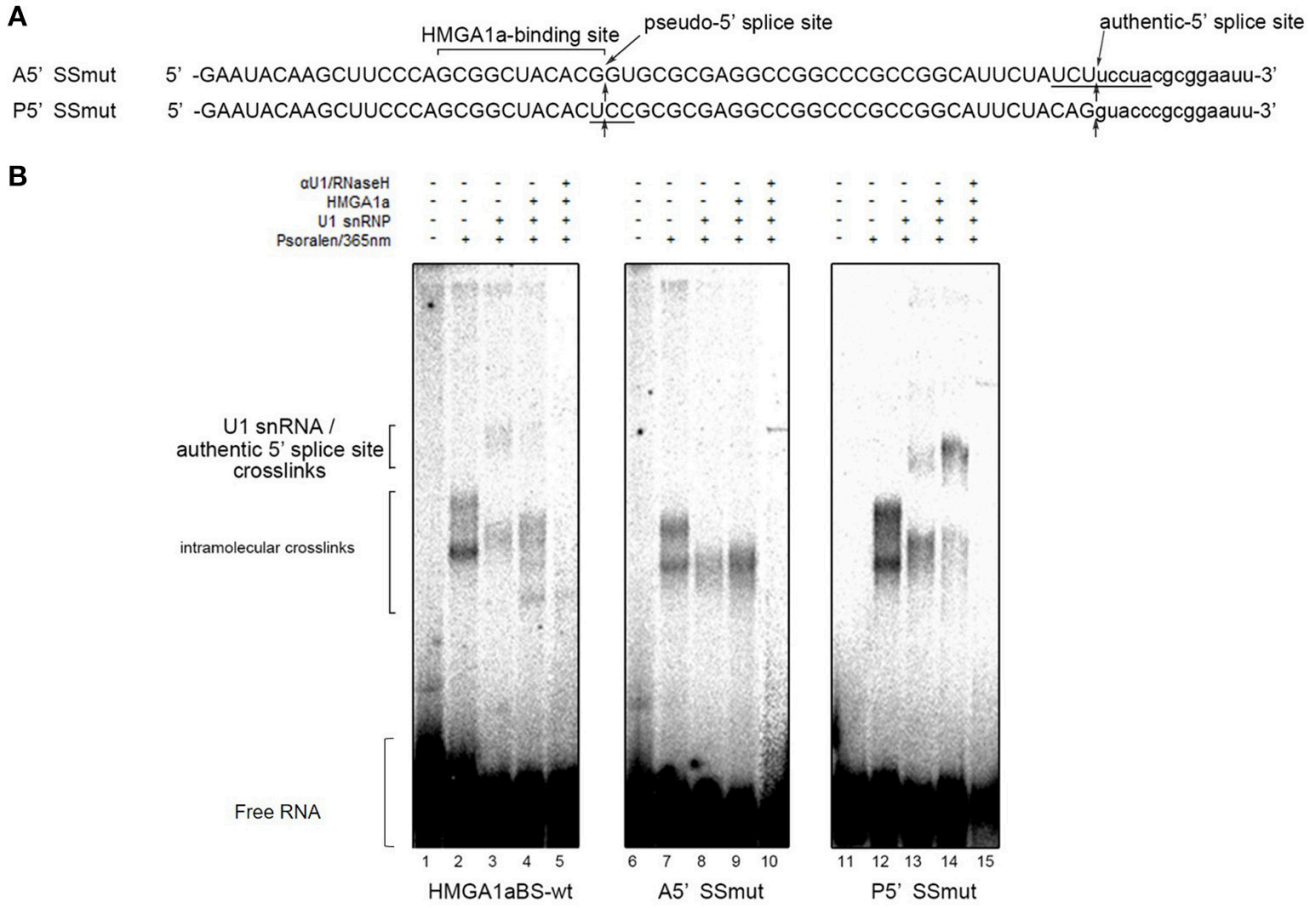
U1 snRNP is anchored to the upstream pseudo-5′ splice site in the presence of HMGA1a. **(A)** Aligned sequences of the short RNAs containing mutated authentic 5′ splice site (Amut) and mutated pseudo-5′ splice site (Pmut). Mutated bases are underlined. Arrows show the pseudo-5′ splice site and authentic-5′ splice site. **(B)** Radiolabeled RNA of **(A)** was crosslinked to purified U1 snRNP (234 ng in 12.5 μl) in the presence (287 ng in 12.5 μl) or absence of HMGA1a by psoralen and 365 nm UV light.

In our previous report, we showed HMGA1a prevents normal dissociation of U1 snRNP from the 5′ splice site only when the 5′ splice site is adjacently downstream the HMGA1a RNA-binding sequence (Ohe and Mayeda, 2010). In the case of *ER*α exon 1, HMGA1a binds adjacently upstream a pseudo-5′ splice site located 33 nucleotides upstream of the authentic splice site (Figure 1). We tested whether HMGA1a could trap U1 snRNP to the pseudo-5′ splice site and block U1 snRNP-binding to the authentic 5′ splice site of *ER*α exon 1 by psoralen-mediated UV crosslinking assay (Figure 3B). We used the same RNA (HMGA1aBS-wt) in the RNA-EMSA experiments (Figure 1A), which contains the pseudo-5′ splice site and authentic 5′ splice site of ERα exon 1. Mutants of each 5′ splice site (Figure 3, A5′SSmut: mutant of authentic 5′ splice site, P5′SSmut: mutant of pseudo-5′ splice site) were used to define each U1 snRNP/5′ splice site crosslink. When purified U1 snRNP was added (5-min incubation), two crosslinks with fast and slow mobility were detected using HMGA1aBS-wt RNA (Figure 3B, lane 3). In the presence of HMGA1a, the fast mobility band increased intensity, while the slow mobility band was almost completely abolished (Figure 3B lane 4). The slow mobility crosslink found in HMGA1aBS-wt could not be detected using A5′SSmut, thus can be designated as the U1 snRNA/authentic 5′ splice site-crosslink. The fast mobility crosslink is difficult to judge because of its similar mobility with the internal crosslinks (Figure 3B, lane 2). This is also the case for A5′SS mut (Figure 3B, compare lanes 8, 9 with lane7) and P5′SS mut (Figure 3B, compare lanes 13, 14 with lane 12). When using P5′SS mut, the intensity of the slow mobility crosslink (U1 snRNA/authentic 5′ splice site) did not decrease, but rather increased in the presence of HMGA1a.

**Figure 4:**
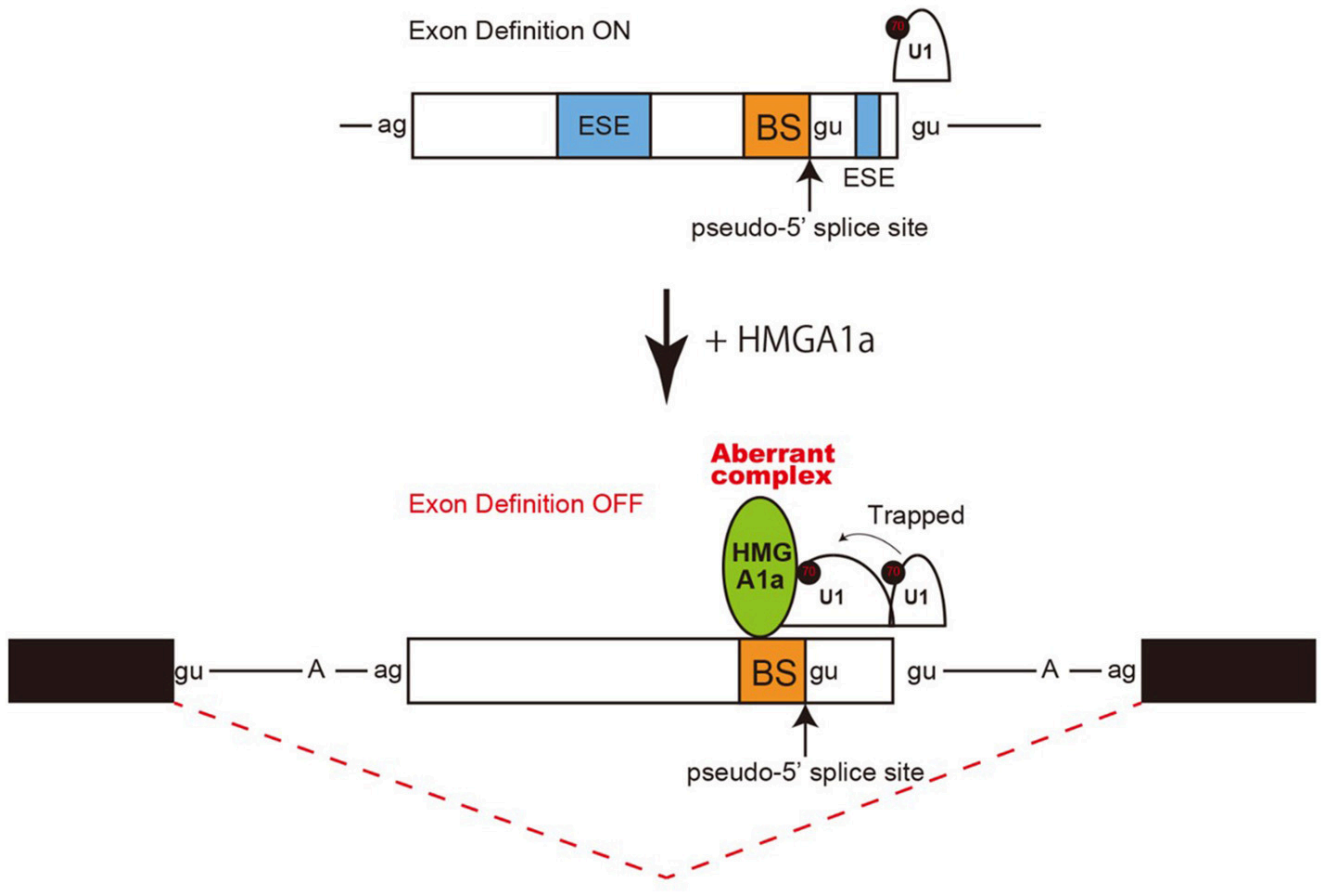
Model and mechanism of HMGA1a-induced ERα alternative splicing. HMGA1a traps U1 snRNP to an upstream pseudo 5′ splice site and disrupts the function of the downstream authentic 5′ splice site.

Taken together, an aberrant complex of HMGA1a-mediated trapping of U1 snRNP to the upstream 5′ splice site inhibited binding of U1 snRNP to the authentic-5′ splice site (Figure 4).

## Discussion

HMGA1a is originally known as a DNA-binding transcription factor but we found it exerts abnormal exon skipping of the *presenilin-2* gene in sporadic Alzheimer's disease through sequence-specific RNA binding to a sequence, 5′-GCUGCUACAAG-3′ (Manabe et al., 2003, 2007; Ohe and Mayeda, 2010). It has also been recently reported of other RNA targets for HMGA1a: regulating Vpr mRNA expression of the HIV gene (Tsuruno et al., 2011); and binding to 7SK snRNA through its DNA-binding domain and thereby affecting its own transcriptional regulation activity (Eilebrecht et al., 2011a,b,c). HMGA1a is known to be multifunctional (Reeves, 2001), with various functions in normal as well as pathophysiological contexts. Indeed, it has been reported as an important component of senescence-associated heterochromatic foci (Narita et al., 2006). This study expands the number of RNA targets of HMGA1a with important pathophysiological function as well as suggesting a novel mechanism in 5′ splice site choice.

Regulation of 5′ splice site function by upstream 5′ splice sites has been analyzed in previous studies and uncovered silencing sequences (Yu et al., 2008) as well as the discovery of upstream 5′ splice sites functioning as enhancers of the authentic 5′ splice site (Hicks et al., 2010). How the intron-proximal 5′ splice site is favored when two comparable 5′ splice sites exist, is not known (Roca et al., 2013). Here we showed an example of such two competing 5′ splice sites in exon 1 of the *estrogen receptor alpha* (*ER*α) gene. The 5′ splice site scores of the two 5′ splice sites are comparable both with a MAXENT score of 8.6. In normal expression of the *ER*α gene the intron-proximal 5′spice site is utilized. Regulation of U1 snRNP binding to alternative 5′ splice sites has been reported to occur during A complex formation, with no difference of U1 snRNP binding to upstream and downstream 5′ splice site in E complex (Hodson et al., 2012). U1 snRNP has been reported to protect a region that is 23 nucleotides upstream into the exon and 12 nucleotides downstream into the intron in PTB-independent conditions (Sharma et al., 2011). Since the two 5′ splice sites of ERα exon 1 are 33 nucleotides apart, the length in-between would allow both 5′ splice sites to bind U1 snRNP but would be in close proximity. We believe the two 5′ splice sites found in *ER*α exon 1 would be a good natural model in studying how the intron-proximal 5′ splice site is favored when the upstream 5′ splice site is in the closest range of simultaneous binding of U1 snRNP. From RNA-EMSA and psoralen crosslinking assays in this study, HMGA1a binded to an upstream sequence adjacent a pseudo 5′ splice site and inhibited U1 snRNP binding to the authentic 5′ splice site only in the presence of the upstream pseudo 5′ splice site. We believe U1 snRNP simultaneously binded to both 5′ splice sites of ERα exon 1 in the absence of HMGA1a, but when HMGA1a is added, U1 snRNP is trapped to form an aberrant complex which inhibits U1 snRNP binding to the authentic 5′ splice site (Figure 4).

However, there is still various limitations in this study. First, if U1 snRNP protection at the upstream 5′ splice site is extended more than U1 snRNP binding in PTB independent conditions (Sharma et al., 2011), U1 snRNP binding to authentic-5′ splice site would be inhibited. This may be the case because we observed an increase of U1 snRNP binding to the authentic 5′ splice site when the upstream 5′ splice site was mutated (Figure 3B, lane 14). An RNase H protection assay with an antisense oligonucleotide directed to check the occupancy of the authentic 5′ splice site would be able to answer this point. Second, there is still a possibility of HMGA1a trapping U1 snRNP to the authentic 5′ splice site. Though we previously found that HMGA1a traps U1 snRNP when the HMGA1a RNA-binding site is adjacent or at least within ten nucleotides of the 5′ splice site (Ohe and Mayeda, 2010), the sequence between the two 5′ splice sites of *ER*α exon 1 is extremely GC-rich (24 out of 32 nucleotides; 75%) and there is a strong possibility of secondary structure leading to close enough range of the HMGA1a RNA-binding site and 5′ splice site for HMGA1a-induced U1 snRNP trapping. This also well explains the increased crosslink of U1 snRNP and the authentic 5′ splice site when the upstream 5′ splice site was mutated (Figure 3B, lane 14).

Besides the precise mechanism of HMGA1a-induced silencing of the downstream authentic 5′ splice site of *ER*α exon 1, this event consequently induced exon skipping and expression of the isoform ERα46. Using decoy RNA that binded to HMGA1a and inhibited HMGA1a-induced exon skipping of ERα, we observed enhanced estrogen-dependent tumor growth and sensitization of tamoxifen-resistant tumor cells to tamoxifen due to increased expression of full length ERα by correction of alternative splicing (Ohe et al., 2018). We hope this HMGA1a-targeted therapy, along with its RNA-binding site, will enlighten a novel strategy in overcoming tamoxifen-resistant breast cancer.

## Author contributions

KO conceived the original idea, conducted experiments (Figures 1B, 2, 3), and wrote the paper. SM, TT, YH, YHa, YHo, YB, FI, TaY, HT, MM, YM, MT, IA, KA, KK, ME, TN, ToY, NH, TU, AM helped conducting the experiments and provided materials as well as advice on the paper. All the authors contributed substantially to the conception or design, data, interpretation of the results, they all drafted and revised the content, and finally approved for publishment. Agreement to be accountable for all aspects of the work in ensuring that questions related to the accuracy or integrity of any part of the work are appropriately investigated and resolved.

### Conflict of interest statement

The authors declare that the research was conducted in the absence of any commercial or financial relationships that could be construed as a potential conflict of interest.

## References

[B1] Barraille P. Chinestra P. Bayard F. Faye J. C. (1999). Alternative initiation of translation accounts for a 67/45 kDa dimorphism of the human estrogen receptor ERalpha. Biochem. Biophys. Res. Commun. 257, 84–88. 10.1006/bbrc.1999.0334 10092514

[B2] Chen J. Weiss W. A. (2015). Alternative splicing in cancer: implications for biology and therapy. Oncogene 34, 1–14. 10.1038/onc.2013.570 24441040

[B3] David C. J. Manley J. L. (2010). Alternative pre-mRNA splicing regulation in cancer: pathways and programs unhinged. Genes Dev. 24, 2343–2364. 10.1101/gad.1973010 21041405 PMC2964746

[B4] Eilebrecht S. Becavin C. Leger H. Benecke B. J. Benecke A. (2011a). HMGA1-dependent and independent 7SK RNA gene regulatory activity. RNA Biol. 8, 143–157. 10.4161/rna.8.1.14261 21282977

[B5] Eilebrecht S. Benecke B. J. Benecke A. (2011b). 7SK snRNA-mediated, gene-specific cooperativity of HMGA1 and P-TEFb. RNA Biol. 8, 1084–1093. 10.4161/rna.8.6.17015 21957495

[B6] Eilebrecht S. Brysbaert G. Wegert T. Urlaub H. Benecke B. J. Benecke A. (2011c). 7SK small nuclear RNA directly affects HMGA1 function in transcription regulation. Nucleic Acids Res. 39, 2057–2072. 10.1093/nar/gkq1153 21087998 PMC3064786

[B7] Filarsky M. Zillner K. Araya I. Villar-Garea A. Merkl R. Langst G.. (2015). The extended AT-hook is a novel RNA binding motif. RNA Biol. 12, 864–876. 10.1080/15476286.2015.1060394 26156556 PMC4615771

[B8] Flouriot G. Brand H. Denger S. Metivier R. Kos M. Reid G.. (2000). Identification of a new isoform of the human estrogen receptor-alpha (hER-alpha) that is encoded by distinct transcripts and that is able to repress hER-alpha activation function 1. EMBO J. 19, 4688–4700. 10.1093/emboj/19.17.4688 10970861 PMC302047

[B9] Hicks M. J. Mueller W. F. Shepard P. J. Hertel K. J. (2010). Competing upstream 5' splice sites enhance the rate of proximal splicing. Mol. Cell Biol. 30, 1878–1886. 10.1128/MCB.01071-09 20123971 PMC2849477

[B10] Hodson M. J. Hudson A. J. Cherny D. Eperon I. C. (2012). The transition in spliceosome assembly from complex E to complex A purges surplus U1 snRNPs from alternative splice sites. Nucleic Acids Res. 40, 6850–6862. 10.1093/nar/gks322 22505580 PMC3413131

[B11] Inoue K. Fry E. A. (2015). Aberrant splicing of estrogen receptor, HER2, and CD44 genes in breast cancer. Genet. Epigenet. 7, 19–32. 10.4137/GEG.S35500 26692764 PMC4669075

[B12] Karni R. de Stanchina E. Lowe S. W. Sinha R. Mu D. Krainer A. R. (2007). The gene encoding the splicing factor SF2/ASF is a proto-oncogene. Nat. Struct. Mol. Biol. 14, 185–193. 10.1038/nsmb1209 17310252 PMC4595851

[B13] Kataoka N. Yong J. Kim V.N. Velazquez F. Perkinson R.A. Wang F.. (2000). Pre-mRNA splicing imprints mRNA in the nucleus with a novel RNA-binding protein that persists in the cytoplasm. Mol. Cell 6, 673–682. 10.1016/S1097-2765(00)00065-4 11030346

[B14] Lund T. Holtlund J. Fredriksen M. Laland S. G. (1983). On the presence of two new high mobility group-like proteins in HeLa S3 cells. FEBS Lett. 152, 163–167. 10.1016/0014-5793(83)80370-6 6297996

[B15] Maasch C. Vater A. Buchner K. Purschke W. G. Eulberg D. Vonhoff S.. (2010). Polyetheylenimine-polyplexes of Spiegelmer NOX-A50 directed against intracellular high mobility group protein A1 (HMGA1) reduce tumor growth *in vivo*. J. Biol. Chem. 285, 40012–40018. 10.1074/jbc.M110.178533 20961861 PMC3000983

[B16] Manabe T. Katayama T. Sato N. Gomi F. Hitomi J. Yanagita T.. (2003). Induced HMGA1a expression causes aberrant splicing of Presenilin-2 pre-mRNA in sporadic Alzheimer's disease. Cell Death Differ. 10, 698–708. 10.1038/sj.cdd.4401221 12761578

[B17] Manabe T. Ohe K. Katayama T. Matsuzaki S. Yanagita T. Okuda H.. (2007). HMGA1a: sequence-specific RNA-binding factor causing sporadic Alzheimer's disease-linked exon skipping of presenilin-2 pre-mRN. Genes Cells A 12, 1179–1191. 10.1111/j.1365-2443.2007.01123.x 17903177

[B18] Mayeda A. Krainer A. R. (1999a). Mammalian *in vitro* splicing assays. Methods Mol. Biol. 118, 315–321. 10549534 10.1385/1-59259-676-2:315

[B19] Mayeda A. Krainer A. R. (1999b). Preparation of HeLa cell nuclear and cytosolic S100 extracts for *in vitro* splicing. Methods Mol. Biol. 118, 309–314. 10549533 10.1385/1-59259-676-2:309

[B20] Narita M. Krizhanovsky V. Nunez S. Chicas A. Hearn S. A. Myers M. P.. (2006). A novel role for high-mobility group a proteins in cellular senescence and heterochromatin formation. Cell 126, 503–514. 10.1016/j.cell.2006.05.052 16901784

[B21] Ohe K. Mayeda A. (2010). HMGA1a trapping of U1 snRNP at an authentic 5' splice site induces aberrant exon skipping in sporadic Alzheimer's disease. Mol. Cell Biol. 30, 2220–2228. 10.1128/MCB.00114-10 20194618 PMC2863597

[B22] Ohe K. Miyajima S. Abe I. Tanaka T. Hamaguchi Y. Harada Y.. (2018). HMGA1a induces alternative splicing of estrogen receptor alpha in MCF-7 human breast cancer cells. J. Steroid Biochem. Mol. Biol. 10.1016/j.jsbmb.2018.04.007 [Epub ahead of print]. 29678492

[B23] Oltean S. Bates D. O. (2014). Hallmarks of alternative splicing in cancer. Oncogene 33, 5311–5318. 10.1038/onc.2013.533 24336324

[B24] Penot G. Le Peron C. Merot Y. Grimaud-Fanouillere E. Ferriere F. Boujrad N.. (2005). The human estrogen receptor-alpha isoform hERalpha46 antagonizes the proliferative influence of hERalpha66 in MCF7 breast cancer cells. Endocrinology 146, 5474–5484. 10.1210/en.2005-0866 16150902

[B25] Pierantoni G. M. Esposito F. Giraud S. Bienvenut W. V. Diaz J. J. Fusco A. (2007). Identification of new high mobility group A1 associated proteins. Proteomics 7, 3735–3742. 10.1002/pmic.200700148 17880001

[B26] Reeves R. (2001). Molecular biology of HMGA proteins: hubs of nuclear function. Gene 277, 63–81. 10.1016/S0378-1119(01)00689-8 11602345

[B27] Reeves R. (2010). Nuclear functions of the HMG proteins. Biochim. Biophys. Acta 1799, 3–14. 10.1016/j.bbagrm.2009.09.001 19748605 PMC2818135

[B28] Roca X. Krainer A. R. Eperon I. C. (2013). Pick one, but be quick: 5' splice sites and the problems of too many choices. Genes Dev. 27, 129–144. 10.1101/gad.209759.112 23348838 PMC3566305

[B29] Sawa H. Ohno M. Sakamoto H. Shimura Y. (1988). Requirement of ATP in the second step of the pre-mRNA splicing reaction. Nucleic Acids Res. 16, 3157–3164. 10.1093/nar/16.8.3157 3375053 PMC336485

[B30] Sgarra R. Furlan C. Zammitti S. Lo Sardo A. Maurizio E. Di Bernardo J.. (2008). Interaction proteomics of the HMGA chromatin architectural factors. Proteomics 8, 4721–4732. 10.1002/pmic.200800193 18850631

[B31] Sgarra R. Tessari M. A. Di Bernardo J. Rustighi A. Zago P. Liberatori S.. (2005). Discovering high mobility group A molecular partners in tumour cells. Proteomics 5, 1494–1506. 10.1002/pmic.200401028 15798993

[B32] Sharma S. Maris C. Allain F. H. Black D. L. (2011). U1 snRNA directly interacts with polypyrimidine tract-binding protein during splicing repression. Mol. Cell 41, 579–588. 10.1016/j.molcel.2011.02.012 21362553 PMC3931528

[B33] Silipo M. Gautrey H. Tyson-Capper A. (2015). Deregulation of splicing factors and breast cancer development. J. Mol. Cell Biol. 7, 388–401. 10.1093/jmcb/mjv027 25948865

[B34] Tsuruno C. Ohe K. Kuramitsu M. Kohma T. Takahama Y. Hamaguchi Y.. (2011). HMGA1a is involved in specific splice site regulation of human immunodeficiency virus type 1. Biochem. Biophys. Res. Commun. 406, 512–517. 10.1016/j.bbrc.2011.02.059 21329653

[B35] Yeo G. Burge C. B. (2004). Maximum entropy modeling of short sequence motifs with applications to RNA splicing signals. J. Comput. Biol. 11, 377–394. 10.1089/1066527041410418 15285897

[B36] Yu Y. Maroney P. A. Denker J. A. Zhang X. H. Dybkov O. Luhrmann R.. (2008). Dynamic regulation of alternative splicing by silencers that modulate 5' splice site competition. Cell 135, 1224–1236. 10.1016/j.cell.2008.10.046 19109894 PMC2645801

